# Nickel Doping Unlocks Ambient-Condition Photostability in Individual Cesium Lead Bromide Perovskite Quantum Dots

**DOI:** 10.1021/acs.nanolett.5c04099

**Published:** 2025-11-12

**Authors:** Jehyeok Ryu, Victor Krivenkov, Adam Olejniczak, Mikel Arruabarrena, Jozef Janovec, Sebastien E. Hadjadj, Maxim Ilyn, Aritz Leonardo, Virginia Martínez-Martínez, Andres Ayuela, Alexey Y. Nikitin, Yury Rakovich

**Affiliations:** † 226245 Donostia International Physics Center (DIPC), Donostia-San Sebastián 20018, Spain; ‡ 202635 Centro de Física de Materiales (CFM-MPC), Donostia-San Sebastián 20018, Spain; § Polymers and Materials: Physics, Chemistry and Technology, Chemistry Faculty, University of the Basque Country (UPV/EHU), Donostia-San Sebastián 20018, Spain; ∥ EHU Quantum Center, University of the Basque Country UPV/EHU, Leioa 48940, Spain; ⊥ Departamento de Química Física, 16402 Universidad del País Vasco, UPV/EHU, Apartado 644, Bilbao 48080, Spain; ▽ IKERBASQUE, Basque Foundation for Science, Bilbao 48013, Spain

**Keywords:** quantum emitter, single-photon source, perovskite quantum dot, photostability, doped perovskite quantum dot

## Abstract

Developing efficient single-photon sources is fundamental to advancing photonic quantum technologies. In particular, achieving scalable, cost-effective, stable, and high-purity single-photon emission at ambient conditions is paramount for free-space quantum communication. However, fulfilling all the requirements simultaneously under ambient conditions has remained a significant challenge. Here we present the scalable, cost-effective ambient condition synthesis of nickel-doped CsPbBr_3_ perovskite quantum dots (NPQDs) using a modified ligand-assisted reprecipitation method. The resulting individual NPQDs demonstrate remarkable photostability, sustaining their performance for over 10 min under ambient conditions, and exhibit exceptional single-photon purity (>99%) with a narrow emission line width (∼70 meV). The remarkable photostability could be attributed to the spatial localization of the exciton by Ni atoms on the surface of the nanocrystal, reducing its interaction with the environment. Our results demonstrated that NPQDs with outstanding combinations of quantum emitting properties can be both synthesized and operated at ambient conditions.

## Graphical abstract

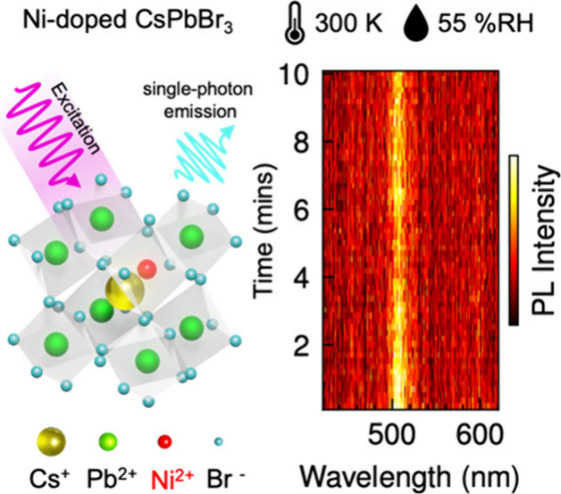

Modern quantum technologies are rapidly evolving, with many of them relying on photon-based qubits at their core. Quantum emitters (QEs) capable of generating individual single-photons are crucial for enabling these flying qubits, serving as the fundamental building blocks for secure quantum communication, as well as quantum metrology, and imaging.^1^ In some quantum key distribution communication protocols like BB84, security relies on high single-photon purity, ensuring that nearly every qubit is encoded to exactly one photon, greatly reducing the risk of multiphoton events that an eavesdropper could exploit.^2^ Similarly, in quantum imaging techniques such as low-light imaging,^3^ the reliability that each detection event corresponds to a single-photon leads to higher image contrast and resolution by reducing false coincidences and background noise. Therefore, high single-photon purity, superior operational stability at ambient conditions are key characteristics required for QEs to meet the demands of the implementation of scalable, cost-effective quantum technologies for real-world use.

Semiconductor quantum dots (QDs) have emerged as promising and efficient quantum emitters.^2^ Notably, epitaxial QDs are already well-established as sources of indistinguishable single-photons at cryogenic temperature.^4^ Nonetheless, practical applications such as free-space quantum communication demand quantum light emission at room temperature or even under ambient conditions,^5^ where epitaxial QDs face challenges in maintaining the single-photon purity and brightness.^6^ In contrast, colloidal QDs can operate as single-photon emitters with the required characteristics even at room temperature. Among them, colloidal lead halide perovskite quantum dots (PQDs) have been intensively explored as QEs at both room and cryogenic temperatures.^7,8^ These PQDs offer near-unity photoluminescence quantum yield (PLQY), high single-photon purity, and considerably longer coherence times compared to other types of colloidal QDs. Furthermore, PQDs exhibit shortened lifetimes^9^ and bright emissive exciton triplet states at low temperature, attributed to inhibited one phonon-assisted exciton transitions to dark singlet ground state,^10,11^ contrary to conventional colloidal QDs. As recently shown by Kaplan et al.,^8^ CsPbBr_3_ PQDs can produce indistinguishable single-photons as verified by Hong–Ou–Mandel interference.

Nevertheless, achieving sufficiently high quality stable PQDs remains a critical step for exploring single-photon emission at the individual QD level. Ligand-assisted reprecipitation (LARP) method provides a scalable, cost-effective synthesis of PQD under ambient conditions, but often produces quantum dots of lower quality with broad size distribution–suitable primarily for optoelectronic applications like solar cells and light emitting diodes. In contrast, hot-injection synthesis typically produces superior-quality monodisperse PQDs but requires elevated temperatures (T: 440–550 K), inert atmosphere, increasing the complexity of the synthetic procedure.^12^ Furthermore, PQDs are structurally labile at the individual nanocrystal level, and prone to degrade under illumination as excess photoexcited charges on the surface react with ambient moisture or oxygen,^13^ limiting their practical applicability as single-photon sources operating at real-world conditions.^14^ To reduce the light-induced surface degradation, strategies to isolate PQDs from moisture and oxygen have been widely explored by encapsulating with polymer^15^ under inert gas^7^ or by engineering ligands for improved passivation.^16^ Zhu et al.^7^ demonstrated that CsPbBr_3_ PQDs encapsulated in a polystyrene matrix layer exhibited photostability for over 80 s in an inert environment, yet the relatively high value of their second-order cross-correlation function *g* ^(2)^(0) ∼ 0.27 indicated nonpure single-photon behavior. Without inert conditions, the PQDs quickly degraded, which was marked by a strong blueshift in their emission spectrum with a further drop in the intensity to zero value. More recently, Morad et al. demonstrated that effective ligand engineering can extend the stable emission of CsPbBr_3_ PQDs to 3 min in an inert environment.^16^ Despite these advancements, achieving stable CsPbBr_3_ PQDs that can operate reliably as single-photon emitters under ambient conditions still remains a major challenge.

An alternative promising strategy for enhancing the stability of PQDs is their doping by transition metal ions.^17^ In particular, doping of CsPbBr_3_ and CsPbI_3_ PQDs, synthesized by hot-injection method, with Ni^2+^ ions has been shown to boost PLQY and environmental stability of PQD ensembles in solutions or dense films.^18–23^ However, for the design of quantum emitters (QEs), such ensemble architectures are unsuitable, as excitation and photon collection must occur from the same individual nanocrystal without interaction with neighboring ones. For quantum-emitting applications, the relevant performance metrics of individual PQDs differ substantially from those of PQD ensembles for LED applications: high single-photon purity, narrow line width, short radiative lifetime, and long-term spectral stability at the single QE level are required. In single QEs, spectral blueshift and size reduction are among the primary mechanisms of photodegradation under ambient conditions, which critically limit their applicability,^7,15,24,25^ while in dense films or colloidal solutions, the predominant stability concern is the loss of ensemble PL intensity.^26^ Thus, despite the demonstrated benefits of Ni^2+^ doping for ensemble PQDs, its specific impact on the quantum-emitting properties of individual PQDs remains largely unexplored.

In this study, we address this critical gap by devising a synthesis protocol for Ni-doped CsPbBr_3_ PQDs (NPQDs). By incorporating Ni^2+^ ions during the glovebox-free LARP synthesis at ambient conditions (room temperature, 70% relative humidity), we obtained highly stable NPQDs solutions with PLQY ∼ 86%. We demonstrated their superior performance by rigorously testing their performance as QEs by evaluating their photostability, photoluminescence (PL) line width, PL lifetime, and single-photon purity under ambient conditions. Notably, this work represents perovskite QDs synthesized entirely under ambient conditions that function as high-purity single-photon sources in the same environment, paving the way for more accessible and scalable quantum technologies.

We synthesized NPQDs by modifying the LARP^27^ method under ambient conditions, as schematically illustrated in Figure 1a. The process involves four key steps: (i) preparation of the seed solution of CsBr, PbBr_2_, and NiBr_2_ (molar ratio 1:1:0.5)^18^ in dimethylformamide (DMF) with oleic acid and oleylamine, followed by sonication for 1 h to effectively dissolve NiBr_2_; (ii) inducing nucleation and formation of nanocrystals by injecting the seed solution into toluene; (iii) ligand exchange from highly dynamic, long-chain ligand (oleylamine) to the more stable, low-steric hindrance, short-chain ligand^28,29^ (phenethylammonium, PEA) to enhance nanocrystal stability; and (iv) purification via centrifugation to obtain monodisperse nanocrystals (see Methods for details). The entire synthesis was performed at room temperature and 70% relative humidity, demonstrating the viability of an ambient-condition synthesis approach. Figure 1b depicts a 3D illustration of the expected crystal structure of a NPQD, where Ni^2+^ ions replace Pb^2+^ ions in the crystal lattice, forming octahedral coordination with halide ions.^20^

**Figure 1:**
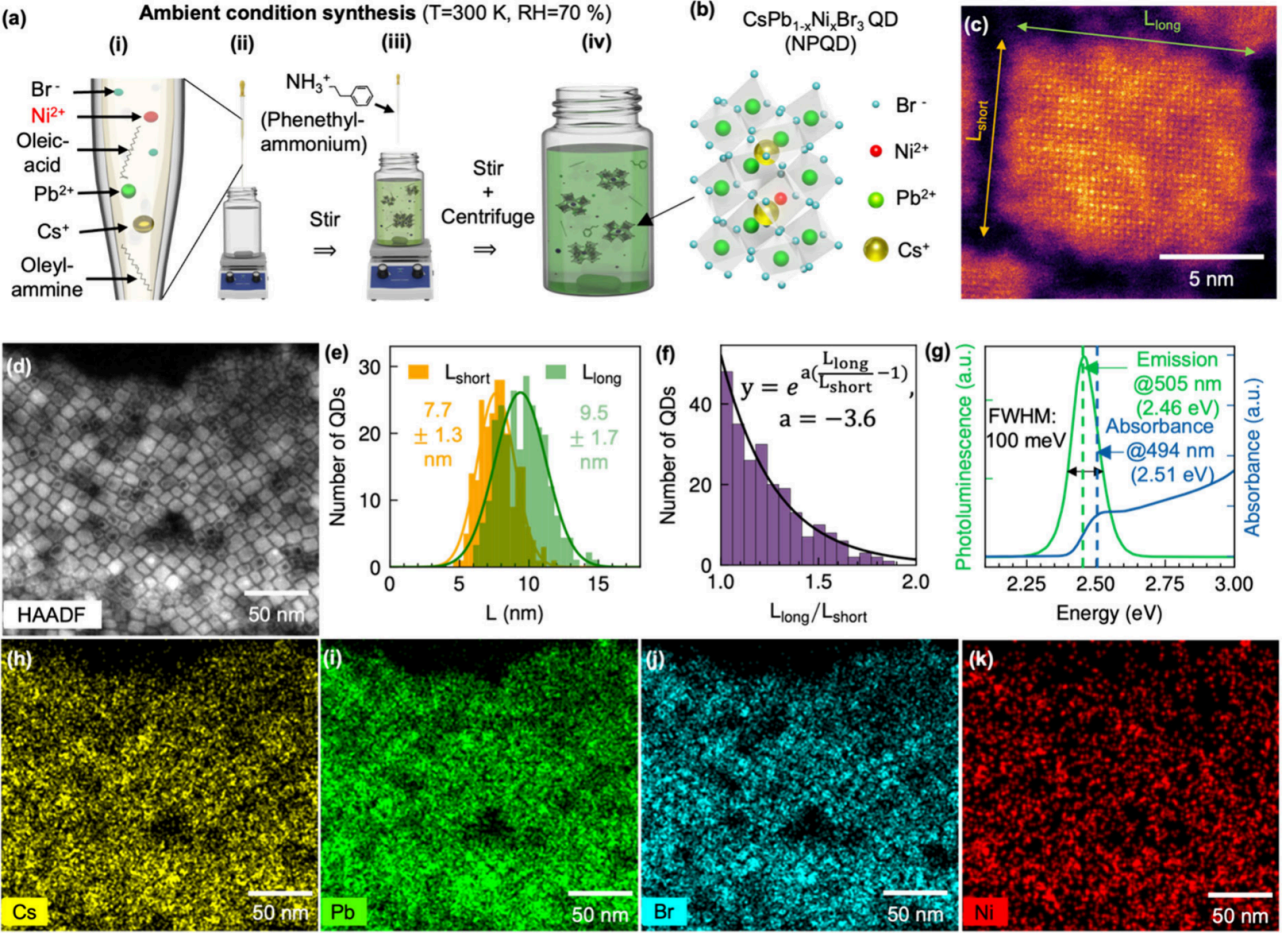
Synthesis and characterization of Ni-doped CsPbBr_3_ PQDs (NPQDs). (a) Schematic illustration of ligand-assisted reprecipitation synthesis under ambient conditions. (b) Schematic crystal structure of NPQD (c, d): HAADF-STEM of single NPQD (c) and NPQDs (d). (e) The distribution of shorter and longer edge lengths (L_short_, and L_long_) of each NPQDs and (f) the histogram of the ratio L_long_/L_short_. (g) Absorbance and PL spectra of NPQD solution. (h, i) The EDS elemental maps of Cs L lines (h), Pb L lines (i), Br K lines (j), and Ni K line (k) (the energies are selected according to Figure S1) in the same scale and area as the HAADF-STEM image (d).

High-angle annular dark field scanning transmission electron microscopy (HAADF-STEM) images (Figure 1c, d) reveal that the synthesized NPQDs retained a cuboid morphology. Notice that dark spots observed on the NPQDs in Figure 1d do not reflect any degradation in their quality as the damages result from the electron beam. To quantify any Ni-induced shape change, we measured the shorter and longer edge lengths (L_short_ and L_long_ in Figure 1c) of each particle in the HAADF-STEM image (Figure 1d) and plotted the distributions in Figure 1e. The distributions of edge lengths are narrow, with mean values of 7.7 ± 1.3 nm for L_short_ and 9.5 ± 1.7 nm for L_long_, indicating that the synthesized NPQDs are monodisperse. A histogram of the aspect ratio L_long_/L_short_ (Figure 1f) shows that cubic particles with aspect ratio near-unity dominate the population and that the number of higher aspect ratio cuboids falls off exponentially. The mean aspect ratio is 1.2, which matches the value reported for pristine CsPbBr_3_ nanocrystals.^30^ These results demonstrate that the introduction of Ni does not significantly alter the particle morphology.

To confirm the incorporation of Ni into the NPQDs, we conducted energy-dispersive X-ray spectroscopy (EDS) spectrum (Figure S1) and elemental maps (Figure 1h-k, Figure S3). Comparison of the HAADF-STEM image (Figure 1d) with the EDS maps shows that Cs, Pb, Br, and Ni signals emanate from the NPQDs themselves. XPS measurements revealed that the sample contained Br, Pb, Cs, and Ni in the correct configurations (Figure S7). The presence of Ni in the nanocrystals is verified with the Ni 2p peaks (Figure S7a). Despite a high molar concentration of Ni during the synthesis, only a small fraction of Ni is incorporated into the nanocrystal, as evidenced by the small molar ratio of Ni/Pb ∼ 0.39% from an independent ICP-AES measurement (Figure S2). This low incorporation level indicates that Ni^2+^ ions tend to be doped rather than forming an alloy. Additionally, selected area electron diffraction (SAED) patterns (Figure S4) show dominant rings corresponding to the (100), (110), and (200) crystal planes of CsPbBr_3_ QDs,^31^ further supporting that the Ni inside the crystal improves crystallinity, which would reduce structural lability.^18^

Optical characterization further evidenced the impact of Ni doping on the optical properties of the NPQD solution. Absorbance and PL spectra of NPQD solution (Figure 1g) show a first exciton peak at 2.51 eV and an emission peak at 2.46 eV with a line width of 100 meV. The NPQD solution exhibits a Stokes shift of about 50 meV, consistent with previously reported values for PQD solutions.^32^ In addition, the emission peak energy of the NPQD solution (Figure S5a) is only slightly blue-shifted, and the PL lifetime of the NPQD solution (Figure S5b) is only modestly shortened compared to the previously reported CsPbBr_3_ solution. This result implies that the exciton–phonon interaction and the energy levels remained similar despite Ni incorporation.

High-purity single-photon emission is a fundamental requirement for quantum photonic technologies, enabling secure quantum communication. However, maintaining photostable high single-photon purity remains a challenge. To assess the emission photostability and quantum emitting characteristics of NPQDs, we prepared thin NPQD-PMMA films with 45 nm thickness (estimated according to ref.^35^), by spin-coating diluted NPQD-PMMA solutions onto glass substrates. PL measurements of individual NPQDs were conducted under ambient conditions (T = 300 K, RH = 55%), as illustrated in Figure 2a.

**Figure 2:**
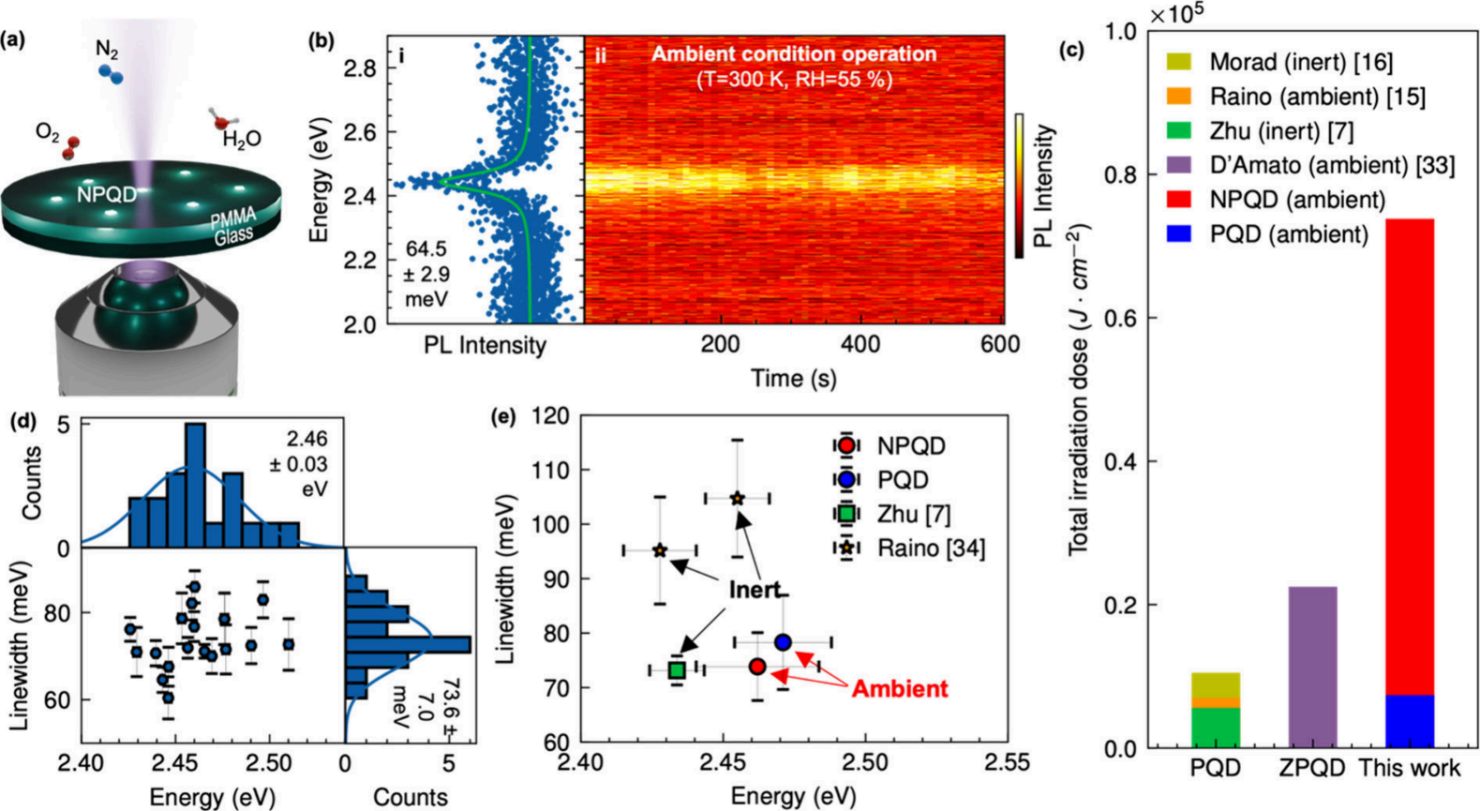
Photostability and PL line widths of individual NPQDs in a PMMA thin film matrix measured under ambient conditions. (a) Schematic illustration of NPQDs embedded in a thin PMMA film spin-coated onto a cover glass. (b) PL spectrum of a representative single NPQD (i) and sequential PL spectrum temporal trace measured for 10 min (ii). (c) Comparison between the studied NPQDs and previously reported values for conventional CsPbBr_3_ QDs^7,15,16,33^ of the total laser irradiation dose before the spectral shift occurred. (d) PL spectrum line width distribution of individual NPQDs and (e) comparison with previously reported values for CsPbBr_3_ PQDs^7,34^

To assess emission photostability, individual NPQDs were excited at 405 nm using a continuous-wave laser source (123 W·cm^–2^). We recorded the PL spectral trace of single NPQDs for 10 min with 10-s photon accumulation time for each measured PL spectrum. This allowed us to monitor the real-time evolution of PL spectra and assess the PL spectral stability. Remarkably, NPQDs displayed narrow line widths down 64.5 meV and remained highly stable for up to 10 min without noticeable spectral shifts and significant intensity loss (Figure 2bii). To compare photostability, we plotted total irradiance dose (averaged power density × irradiated time, J·cm^–2^), representing total energy exposure. Previous studies have reported that undoped 10 nm CsPbBr_3_ PQDs embedded in polystyrene films sustained emission for up to only 100 s at 70 W·cm^–2^ under ambient condition,^15^ corresponding to 7000 J·cm^–2^ irradiation dose (orange bar in Figure 2c), thus demonstrating a low photostability. In an inert atmosphere, emission improved but remained below 200 s as indicated by green (5600 J·cm^–2^) and yellow bar (10500 J·cm^–2^) in Figure 2c.^7,16^ Zn-doped CsPbBr_3_ PQDs have been reported to maintain emission for 60 min at 6.5 W·cm^–2^, corresponding to 22500 J·cm^–2^ of irradiation dose, demonstrating improved photostability via metal doping^33^ (purple bar in Figure 2c). Notably, our NPQDs exhibit an irradiation dose of 73800 J·cm^–2^, indicating a dramatic extension of photostability (red bar in Figure 2c), suggesting that Ni incorporation effectively suppresses common degradation pathways, and highlighting their potential for practical single-photon applications. The corresponding comparison of photostability data reported in the literature for individual CsPbBr_3_ PQDs could be found in Table S1 in the Supporting Information file.

Furthermore, we investigate the emission line widths of individual NPQDs, as it is a direct indicator of dephasing. Line width broadening is mainly driven by exciton–phonon coupling and interactions with the environment. To assess line width distribution, we plotted the first 10-s accumulated PL spectra of 20 individual NPQDs (Figure 2d). The statistical distribution of line widths follows a Gaussian distribution with a mean value of 73.6 ± 7.0 meV, and emission peaks centered at 2.46 ± 0.03 eV. Remarkably, the line width of NPQDs is similar to that of high-quality undoped 20 nm CsPbBr_3_ PQDs, which highlights the excellent optical performance of our QDs at room temperature.^8^ Furthermore, comparing line widths as a function of emission energy reveals that NPQDs operating under ambient conditions (red circle in Figure 2e) have line widths comparable to the best values reported for CsPbBr_3_ PQDs operated under an inert environment (green square in Figure 2e).^7^

We hypothesize that the enhanced photostability and narrower line width observed in individual NPQDs arise from the incorporation of Ni into the nanocrystal, which reduces exciton–environment interactions. To support this hypothesis, we performed density functional theory (DFT) calculations of the crystal formation energies for both pristine and Ni-doped bulk CsPbBr_3_ to investigate the most favorable position of the Ni atom within the crystal. The calculations indicate that both pristine and Ni-doped CsPbBr_3_ are thermodynamically stable, confirming that Ni atoms can indeed be incorporated in the crystal (Figure S8a). Nevertheless, the formation energy of the Ni-doped CsPbBr_3_ is noticeably higher than that of pristine CsPbBr_3_ (see Supplementary Notes (SN) in the Supporting Information for the details), implying that Ni atoms energetically tend to migrate to the surface (Figure S8b).

To understand how the Ni atoms affect the band-edge exciton, we studied the charge density of the latter for both pristine and Ni-doped bulk CsPbBr_3_ (Figure S9c, d, see SN for the details). In pristine CsPbBr_3_, the band-edge exciton is delocalized across the lattice, predominantly around Pb atoms, implying a high probability of interaction between the exciton and the environment (Figure S9c). Even though the exciton in the Ni-doped CsPbBr_3_ is delocalized across the lattice around Pb atoms, it is unconfined to Ni atoms (Figure S9d). Hence, we anticipate that the exciton in the Ni-doped CsPbBr_3_ nanocrystal is unconfined to Ni atoms at the surface, reducing the interaction with the environment. Furthermore, we calculated the defect formation energies for both pristine and Ni-doped CsPbBr_3_ to evaluate whether Ni incorporation reduces defect formation (Figure S11). The DFT results show that the formation energy of the bromide vacancies is comparable, or even slightly lower, in Ni-doped CsPbBr_3_ compared to pristine CsPbBr_3_. This indicates that Ni doping does not lead to reduced defect formation. Therefore, we conclude that the enhanced photostability observed in Ni-doped CsPbBr_3_ is not related to the defect passivation. In brief, we speculate that our DFT calculations provide a hint that the spatial localization of the band edge exciton in the presence of Ni atoms can be a path to improve photostability by reducing interaction with the environment.

Our experimental and theoretical analyses demonstrate that Ni-doping not only enhances the operational stability of NPQDs under ambient conditions but also maintains their remarkable optical quality, making them promising for practical quantum photonic applications.

To assess the single-photon emission characteristics of NPQDs, we performed Hanbury-Brown and Twiss (HBT) measurements, which provide insights into photon statistics from individual NPQDs (Figure 3a). Using a pulsed laser at 405 nm with a 5 MHz repetition rate, ∼200 ps pulse duration, and 31 W cm^–2^ average intensity, we excited NPQDs embedded in PMMA on glass substrates. Due to demonstrated high photostability over 200 s for all individual NPQDs (Figure S6), we could conclude that the g^(2)^ measurements, performed only during 100 s, were unaffected by the photoinduced spectral blue-shift, which could be attributed to the size shrinkage and thus the change of the single-photon purity properties, in contrast to previously reported results for CsPbBr_3_ PQDs.^7,13^

**Figure 3:**
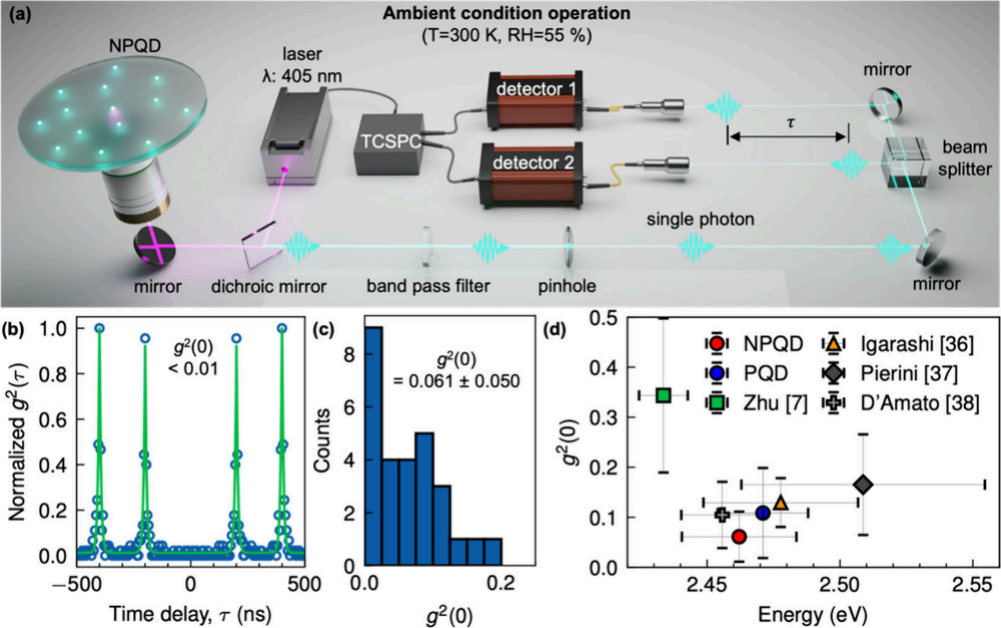
Single-photon purity of an individual NPQD. (a) Schematic illustration of Hanbury-Brown and Twiss setup for verification of single-photon purity (*g* ^(2)^(τ) measurements). (b) *g* ^(2)^(τ) of the representative NPQD. (c) Statistics of *g* ^(2)^(0) values of NPQDs and (d) their comparison with previously reported values for CsPbBr_3_ PQDs.^7,36–38^

The second-order correlation function, *g* ^(2)^(τ), was reconstructed from a time-tagged time-resolved photon arrival data array without any background noise subtraction, for 30 individual NPQDs. Notably, our best-performing individual NPQDs exhibited *g* ^(2)^(0) < 0.01 under ambient conditions without any temporal or spectral filtering (Figure 3b), indicating high-purity single-photon emission from synthesized nanocrystals. Among the 30 individual NPQDs tested, all exhibited *g* ^(2)^(0) < 0.2, with over 70% displaying values below 0.1, with a mean value *g* ^(2)^(0) = 0.061 ± 0.050 (Figure 3c). This high level of single-photon purity rivals even epitaxial quantum dots, which often require cryogenic conditions for comparable performance. These findings underscore the strong potential of NPQDs as a highly promising platform for practical, scalable quantum photonics. In Figure 3d, we compare the mean *g* ^(2)^(0) value measured from our NPQDs with previously reported values measured at room temperature.^7,36–38^ Notably, our values surpass previously reported values of CsPbBr_3_ PQDs. Specifically, photostable individual 10 nm CsPbBr_3_ PQDs in an inert atmosphere (green square in Figure 3d) have a rather low single-photon purity (*g* ^(2)^(0) > 0.3),^7^ indicating insufficient suppression of multiexciton generation at this size. Besides, the three points (gray, orange, black points) in Figure 3d show relatively low *g* ^(2)^(0) values (0.1–0.2), however details about the operating atmosphere condition and photostability were not explicitly provided. It is known that relatively low *g* ^(2)^(0) values can result from increased confinement due to size shrinkage by photodegradation.^7^ In contrast, our individual NPQDs exhibit high single-photon purity while maintaining their photostability, demonstrating their feasibility as efficient single-photon sources. The corresponding comparison of single-photon purity data reported in the literature for individual CsPbBr_3_ PQDs could be found in Table S1 in the Supporting Information file.

We hypothesize that the nonzero *g* ^(2)^(0) values in our NPQDs (indicating the nonideal single-photon emission) arise from the multiphoton emission from NPQDs themselves and not from the environment. Indeed, biexciton–exciton cascades can lead to two-photon emission in a quantum dot.^39^ To verify the origin of nonzero *g* ^(2)^(0) values in some of the NPQDs, we performed time-gating analysis of the emission signal (Figure 4a). In this technique, multiphoton emissions that came in a certain time range after the excitation pulse are excluded. As illustrated in the top part of Figure 4a, two photons can be registered after a laser pulse. However, with time gating (bottom of Figure 4a), certain time windows from each laser pulse are excluded, maintaining pure single-photon statistics. In CsPbBr_3_ PQDs, the biexciton–exciton recombination occurs typically on the order of tens of picoseconds,^39^ which is significantly faster than exciton-ground recombination decay, occurring on the order of a few nanoseconds. Thus, if the main reason for the nonzero *g* ^(2)^(0) level is the biexciton emission, then the time gating technique should reduce this level to near zero. Indeed, by applying a 1 ns time gate which selectively excludes early arriving photons associated with biexciton recombination (inset in Figure 4c), we were able to effectively isolate single-photon emission so that the *g* ^(2)^(0) value for a representative NPQD was reduced from approximately 0.092 (Figure 4b) to less than 0.01 (Figure 4c). To confirm the improvement in single-photon purity for the whole set of NPQDs after time-gating, we plotted *g* ^(2)^(0) values against t_short_, where t_short_ is the short-lifetime component obtained from a typical biexponential lifetime fit (Figure 4d). Before applying time-gating, the *g* ^(2)^(0) values tended to increase at shorter t_short_ due to increased contributions from photons originating from biexcitons (Figure 4d, top). However, a significant percentage of NPQDs exhibited *g* ^(2)^(0) values reduced to less than 0.01 after time gating (Figure 4d, bottom), supporting our hypothesis that the main contribution to nonzero *g* ^(2)^(0) value is the emission from the biexciton–exciton transition. These results demonstrate that Ni doping significantly enhances the single-photon purity of CsPbBr_3_ PQDs, achieving high single-photon purity while maintaining exceptional photostability under ambient conditions. The combination of high single-photon purity, extended emission stability, and the ambient-compatible synthesis positions NPQDs as promising candidates for practical quantum communication and photonic circuit applications.

**Figure 4:**
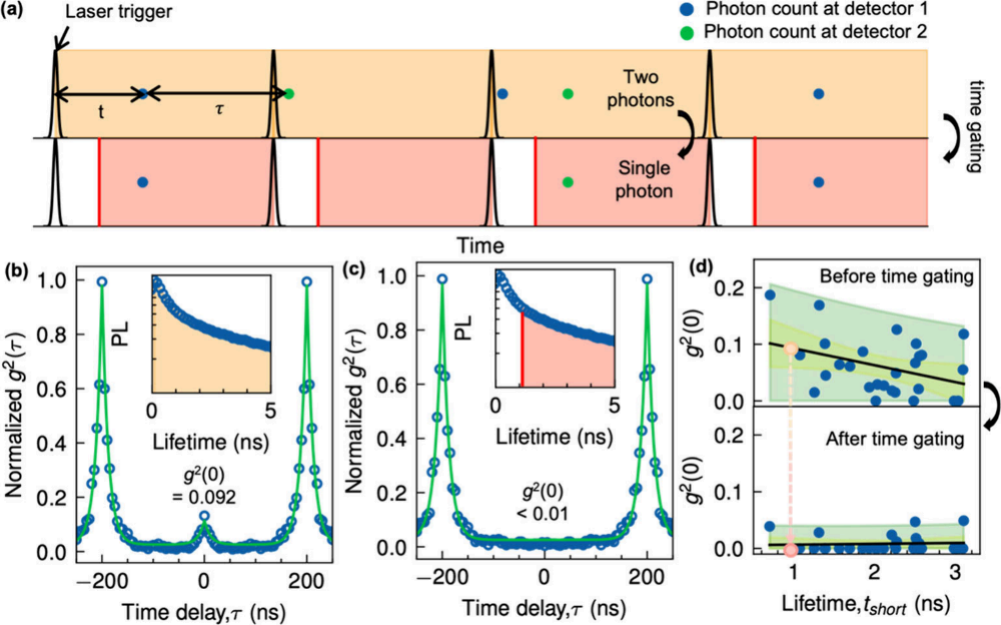
Time-gated single-photon purity of individual NPQDs. (a) Schematic illustration of the time-gating process. (b) Normalized *g* ^(2)^(τ) of the representative individual NPQD before time-gating, which corresponds to the apricot circle in (d); (c) the same after time-gating, which corresponds to the red circle in (d). (d) *g* ^(2)^(τ) value statistics of individual NPQDs before (top) and after (bottom) time-gating.

Thus, we have overcome significant barriers in the development of high-performance single-photon quantum sources by demonstrating a straightforward method to synthesize high-quality perovskite quantum dots entirely under ambient conditions. The introduction of Ni doping has not only enhanced the photostability of the CsPbBr_3_ PQDs but also enabled single-photon emission with exceptional purity exceeding 99%. This success allows the development of stable and efficient single-photon sources based on NPQDs integrated into real devices without the need for a controlled environment. The cost-effectiveness and simplicity of the synthesis process open up opportunities for a broad range of applications, including quantum communications and cryptography, photonic circuits, and optoelectronic devices where stability under ambient conditions is paramount. Moreover, the demonstrated ability to fabricate high-quality NPQDs in thin film lays the groundwork for coupling them with photonic cavities and exploring their integration into quantum photonic circuits.

## Supplementary Material



## Data Availability

The data that support the findings of this study are available from the corresponding author upon reasonable request.

## Notes

EDS spectra and image map, ICP-AES measurements, SAED, and photostability spectra of the Ni-doped CsPbBr_3_ QDs; DFT calculation (PDF)
