# Supplementary material for: Nickel Doping Unlocks Ambient-Condition Photostability in Individual Cesium Lead Bromide Perovskite Quantum Dots
Source: Nano Lett. 2025 Nov 12;25(47):16630–6. doi: 10.1021/acs.nanolett.5c04099 (PMC12670497; doi:10.1021/acs.nanolett.5c04099)
Supplement: Supplementary file 1 [file nl5c04099_si_001.pdf]

Supporting Information

Nickel Doping Unlocks Ambient-Condition Photostability in Individual Cesium Lead Bromide Perovskite Quantum Dots

Jehyeok Ryu[1,3,‡], Victor Krivenkov[2,3,‡], Adam Olejniczak[2], Mikel Arruabarrena[1,2], Jozef Janovec[1,2,3], Sebastien E. Hadjadj[2], Maxim Ilyn[2], Aritz Leonardo[1,4], Virginia Martínez-Martínez[5], Andres Ayuela[1,2], Alexey Y. Nikitin[1,6], Yury Rakovich[1,2,3,6*]*

1. Methods

1.1 Synthesis of Ni-Doped $CsPbBr_3$ Quantum Dots

Ni-doped $CsPbBr_3$ NCs were synthesized using a Ligand-Assisted reprecipitation (LARP) method under ambient conditions. First, 17.9 mg of CsBr (99.999%, Sigma-Aldrich) was dissolved in 2 mL of dimethylformamide (DMF, anhydrous, Sigma-Aldrich) and sonicated at room temperature for 2 hours until fully dissolved. Then, 30.8 mg of $PbBr_2$ (99.999%, Sigma-Aldrich) was added, and the mixture was sonicated for an additional 10 minutes to achieve a transparent solution.

After dissolving CsBr and $PbBr_2$ in DMF, 9.2 mg of $NiBr_2$ (99.999%, Sigma-Aldrich) was introduced to the solution, maintaining a molar ratio of $NiBr_2$:$PbBr_2$ = 1:2. The mixture was sonicated for 1 hour, resulting in a yellow ochre solution. Next, 200 µL of oleic acid (technical grade, 90%) and 100 µL of oleylamine (> 98%, primary amine, Sigma-Aldrich) were added while stirring vigorously for 10 minutes, followed by 1 hour of sonication. 40 µL of this seed solution was injected into 2 mL of anhydrous toluene, followed by continuous stirring for 2 hours. The resulting solution turned green-yellowish, indicating successful nanocrystal formation. To

passivate the nanocrystals, 2.5 μL of a 200 mg/mL phenethylammonium bromide (PEABr, >98%, Sigma-Aldrich) solution in DMF was added, and the mixture was stirred for an additional 2 hours. The solution was left to sit for 3 days before being centrifuged at 9000 rpm for 5 minutes. The supernatant was then collected and diluted 3 to 4 times to achieve an absorbance of 0.2 at the first exciton level.

1.2 Preparation of Spin-Coated Film Samples for Single QD Investigation

Spin-coated films for single quantum dot (QD) investigation were prepared by diluting 5 μL of the QD solution in 395 μL of a 1 wt% polymethyl methacrylate (PMMA) solution in anhydrous toluene. Round cover glass substrates (VWR) were annealed at 445°C for 30 minutes to remove any organic contaminants. After annealing, 400 μL of the diluted QD/PMMA/toluene solution was dispensed onto the surface of the cover glass, ensuring complete coverage. The sample was then spin-coated under ambient conditions at 1000 rpm for 1 minute, resulting in a uniform thin film (thickness ~45 nm). The resulting film was immediately ready for single QD investigation. We could find bright and stable individual PQDs during the week after preparation.

1.3 Photoluminescence measurement for individual NPQDs

We used a MicroTime 200 inverted microscope (PicoQuant) with a water immersion objective lens (NA=1.2) to study emission properties from a single NPQD. To measure time-dependent photoluminescence (PL) spectra, individual NPQDs embedded in a thin PMMA layer on a glass substrate were excited using a continuous wave 405 nm laser at an intensity of 123 $W \cdot cm^{-2}$. The PL spectra of single NPQDs were acquired over a 10 minutes period, with each spectrum accumulated for 10 s detected by a charged coupled device (CCD) camera (Andor Kymera 193i Newton spectrometer). The emitted photons passed through a set of filters selecting the 470-550

nm spectral range and passed through a 150 μm pinhole. Under these conditions, the PL signals were typically recorded at a count rate of 20−30 kcps.

To study single-photon purity, we employed a Hanbury Brown Twiss (HBT) set-up, as schematically illustrated in Fig.3a. NPQDs embedded in PMMA on glass substrates were excited by a pulsed 405 nm laser with a 5 MHz repetition rate, ~200 ps pulse duration, and an average intensity of 31 $W \cdot cm^{-2}$. The emitted photons were filtered to select the 470-550 nm spectral range and passed through a 50 μm pinhole. The resulting signal was split by a 50/50 beam splitter and detected by two single-photon avalanche detectors (SPADs). Under these conditions, the PL signal was typically recorded at a count rate of 10−15 kcps.

1.4 Density functional theory calculations

To theoretically investigate the $CsPbBr_3$ perovskites, we performed density functional theory (DFT) calculations using the Vienna Ab-initio Simulation Package (VASP)NS. The projector augmented wave (PAW) method was employed with the Perdew-Burke-Ernzerhof (PBE) generalized gradient approximation (GGA) for the exchange-correlation potential. A GGA+U approach, following Dudarev's formulation[1] was used to account for the on-site Coulomb interactions of the Ni d states. The valence electron configurations considered were $5s^2 5p^6 6s^1$ (Cs), $5d^{10} 6s^2 6p^2$ (Pb), $3p^6 3d^9 4s^1$ (Ni) and $4s^2 4p^5$ (Br).

All calculations were performed with well-converged parameters: an electron plane-wave cutoff energy of 850 eV, a 4×4×4 Γ-centered Monkhorst-Pack k-point mesh, and a Fermi level smearing of 0.1 eV. The electronic energy convergence criterion was set to 10^{-8} eV, and atomic coordinates were relaxed until forces on all atoms were less than 1 meV/Å. Ni doping was simulated by substituting a Pb atom with Ni in a 1×1×2 orthorhombic supercell containing 40 atoms. The relaxed

lattice parameters for the pristine and doped structures were a = 8.27 Å, b = 8.53 Å, c = 23.80 Å and a = 8.21 Å, b = 8.3 Å, c = 23.69 Å, respectively.

For the GW calculations supporting Figure S8, a setup of 500 eV cutoff and 90 frequency points in combination with 512 unoccupied bands was used. The optical absorption spectrum was calculated by the Bethe-Salpeter equation within the Tamm-Dancoff approximation [2,3], using 26 energy dispersion branches within valence and conduction bands, with a maximum transition energy of 12 eV, with a 3x3x3 k-point mesh.

1.5 Transmission electron microscopy

HAADF-STEM and EDX, SAED measurements were carried out using a FEI Titan Cubed G2 60–300 microscope equipped with a Schottky X-FEG field emission source, operated at 80 kV to minimize beam-induced damage to the perovskite quantum dots.

HAADF-STEM imaging was performed with a camera length of 91 mm to enhance Z-contrast by collecting electrons scattered at higher angles. The electron dose was limited to ~33 $e^{-}/Å^{2}\cdot s$ during imaging.

EDX spectra and elemental maps were collected from ensembles of Ni-doped $CsPbBr_3$ quantum dots using a Bruker EDX detector (Esprit 1.9 software). The detector was operated at 80 kV with a take-off angle of 18°, azimuth angle of 45°, and tilt angle of 0°. The Ni signal was identified using the Ni-K line.

Selected area electron diffraction (SAED) was performed in TEM mode at 80 kV using a selected area aperture to isolate individual or clustered quantum dots. The resulting diffraction patterns were used to confirm the crystalline structure of the perovskite phase.

Inductively coupled plasma atomic emission (ICP-AES)

The nickel content in the Ni-doped $CsPbBr_3$ quantum dot (NPQD) samples was quantified using an inductively coupled plasma atomic emission spectrometer (ICP-AES; Optima 8300, PerkinElmer). The instrument provides sufficient sensitivity for Ni detection, as the measured concentration of Ni in the sample (0.22 ppm) is well above the calibration baseline (0.01 ppm).

To prepare the sample for ICP-AES, 40 mL of the NPQD stock solution was first precipitated by the addition of ethanol, followed by centrifugation. The resulting sample was dissolved in a solution of 9.7 mL of deionized water and 0.3 mL of HNO_3. The acidic medium ensures full dissolution and stabilization of Ni ions in solution.

X-ray photoelectron spectroscopy (XPS)

X-ray photoelectron spectroscopy (XPS) measurements were performed using a non-monochromatic X-ray source and a Phoibos 100 analyzer with an energy resolution of ~0.1 eV. All measurements were performed under ultra-high vacuum (UHV) conditions ($1x10^{-9}$ - $5x10^{-10}$ mbar) using the Al anode (Kα radiation). To remove environmental contamination, the sample was sputtered using 500 V and 10 mA for three minutes.

The XPS measurements revealed, that the sample contained Br, Pb, Cs, and Ni in the correct configurations. Since the dropcast sample was deposited on a SiO_2 substrate, an additional shift of around 4-5 eV to higher binding energies must be considered. The Cs 3d, Pb 4f, Si 2p, and Br 3p core-level spectra exhibit spin-orbit splitting, causing specific intensity ratios of 2:1, 3:2, and 4:3 (p, d, and f orbitals), while maintaining an identical full width at half maximum (FWHM) for the two peaks. The additional side peaks (shifted 9.8 eV to lower binding energies) are artifacts of the

non-monochromatic X-ray gun (Al anode) and are unrelated to secondary electron processes such as Auger processes. The Ni 2p peaks prove that the sample contains low concentrations of Ni.

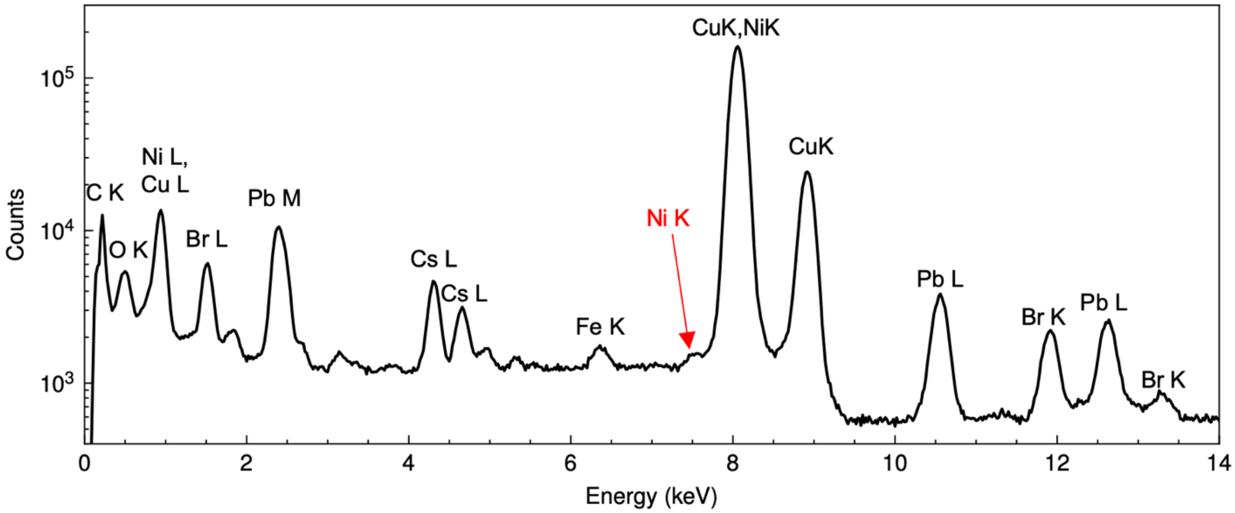

Figure S1. Energy-dispersive X-ray spectroscopy (EDS) of NPQDs. The red arrow marks the Ni K-line signal. Other possible Ni-related peaks overlap with the Cu signal from the TEM grid, making them difficult to resolve.

Element (X)	*Ppm (mg/L)*	*X/Pb molar ratio*
Nickel	*0.22*	*0.39 %*
Lead	*198*	*100%*

Figure S2. Molar ratio of Ni in NPQD samples. The relative concentrations of Ni and Pb were determined by ICP-AES (see Methods), confirming the successful incorporation of Ni into the quantum dots. The Ni concentration (0.22 ppm) is significantly above the detection threshold (0.01 ppm), indicating the instrument's sufficient sensitivity for quantification.

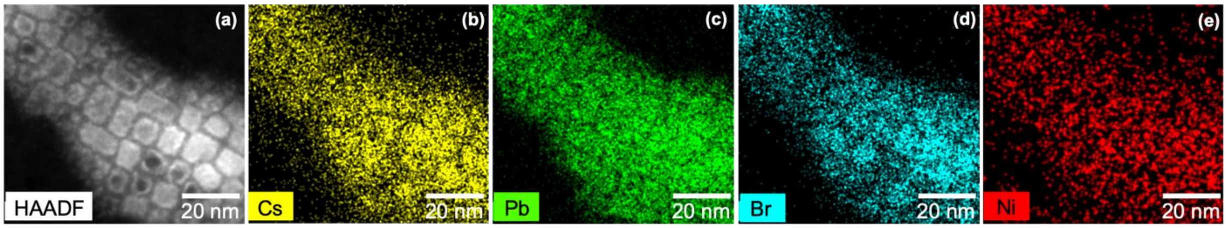

Figure S3. The HAADF-STEM image and the EDS elemental maps of NPQDs. (a) HAADF-STEM image of NPQD ensembles. (b-e). The EDS elemental maps of NPQDs of Cs (b, yellow), Pb (c, green), Br (d, cyan), Ni (e, red). All the elemental signals were confirmed to originate from the NPQD samples. The Ni signal was obtained from the Ni K-line, as indicated by the red arrow in Figure S1.

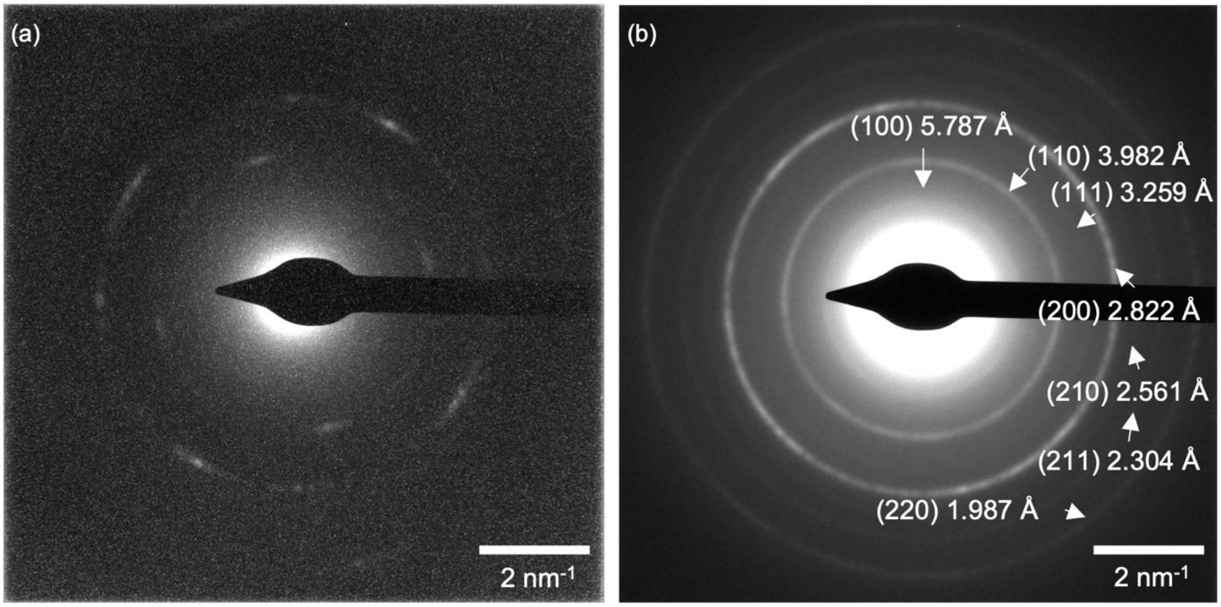

Figure S4. Enhanced crystallinity of NPQDs compared to pristine PQDs. Selected area electron diffraction (SAED) patterns of (a) pristine PQDs and (b) Ni-doped PQDs. Patterns were recorded from ensembles in each case. The NPQDs exhibit clearer and more intense diffraction rings corresponding to various crystal planes ((100), (110), (111), (200), (210), (211), (220)), indicating improved crystallinity after Ni incorporation.

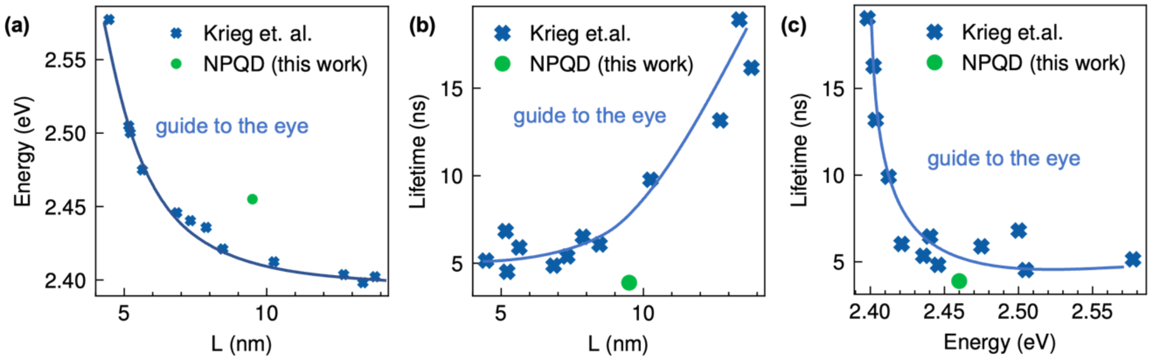

Figure S5. Comparison of emission peak and lifetime of perovskite QD solution. (a, b) Size-dependent emission peak (a) and lifetime (b) of perovskite QD solution. (c) Energy-dependent lifetime of perovskite QD solution. Blue dots are values of pristine $CsPbBr_3$ QD solution extracted from the literature[4]. Adapted with permission from Krieg et al., ACS Cent. Sci. **7**, 135 (2021). Copyright 2020 American Chemical Society. Green dots are the size-dependent emission peak of the Ni-doped $CsPbBr_3$ QD solution from this work.

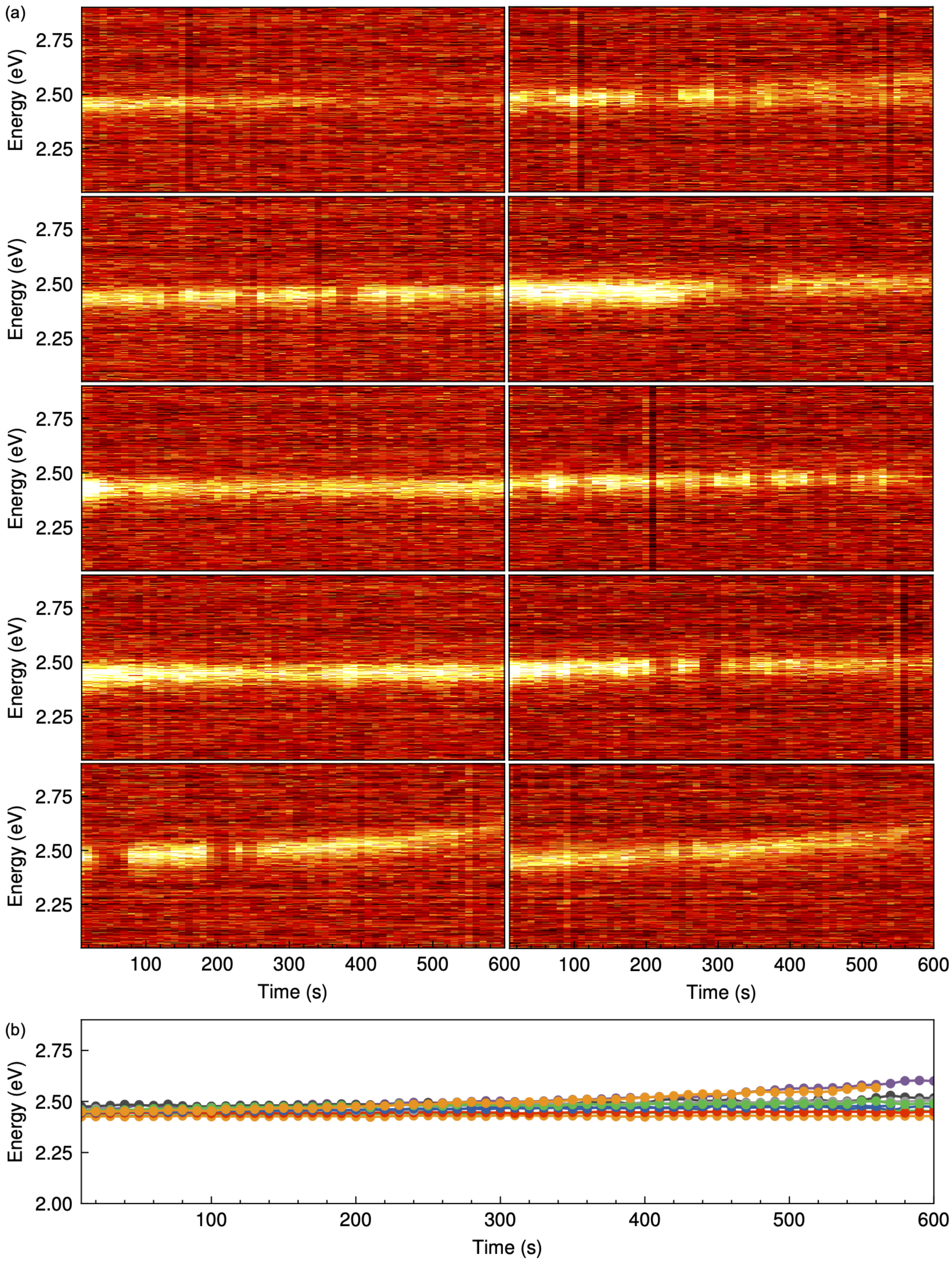

Figure S6. Photostability of individual NPQDs. (a) Time-dependent spectra of individual NPQDs. (b) Time trace of emission peak positions extracted from panel (a).

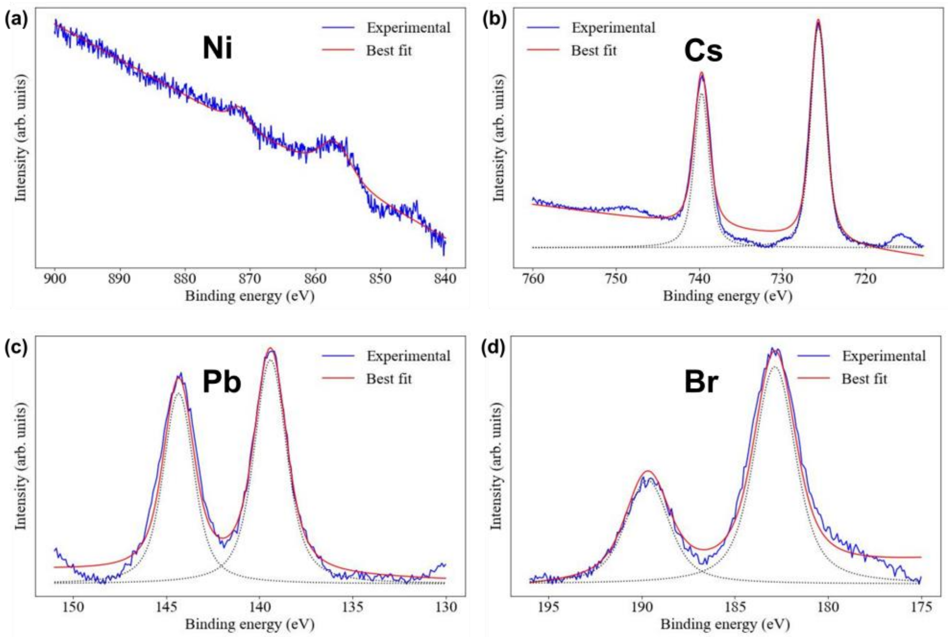

Figure S7. Results of XPS measurements. Peaks relate (a) Ni peaks; (b) Cs peaks; (c) Pb peaks; (d) Br peaks.

Table S1. Reported single-photon performance metrics of $CsPbBr_3$ nanocrystals.

Source	$g^2(0)$	Linewidth, meV	Total irradiation dose ($J\cdot cm^{-2}$)	Measurement conditions	Synthesis conditions
PQD (this work)	0.1085	78.3	7380	Ambient	Ambient conditions synthesis
NPQD (this work)	0.061	73.86	73800	Ambient	Ambient conditions synthesis
Zhu et al. Ref.[5]	0.345	73.15	5600	Inert atmosphere	Hot injection
Igarashi et al.[6]	0.129	Unknown	Unknown	Unknown	Hot injection
Pierini et al.[7]	0.165	>71.5	Unknown	Unknown	Hot injection
D'Amato et al.[8]	0.104	72.19	Unknown	Unknown	Hot injection
Morad et al.[9]	0.11	Unknown	10500	Inert atmosphere	Ambient conditions synthesis
D'Amato et al.[10]	>0.1	~70	22500	Ambient	Hot injection
Raino et al.[11]	~0.1	Unknown	7000	Ambient	Hot injection

2. Supplementary Notes

The interaction of the hot electron from the exciton with the nanocrystal surface is one of the primary factors contributing to nanocrystal photodegradation and photochemical etching caused by oxygen and water molecules from the environment [12–14]. We hypothesized that doping PQDs with Ni ions can diminish this interaction, thereby improving the stability of NPQDs.

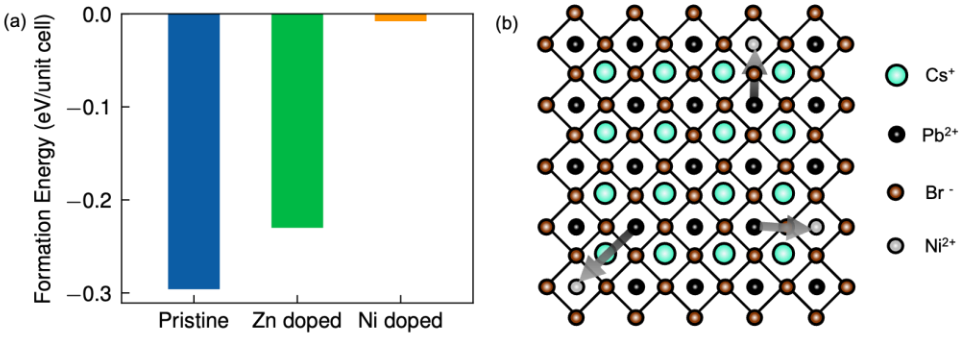

Figure S8. Crystal formation energy of pristine, Zn-doped, and Ni-doped $CsPbBr_3$. (a) The blue, green, and orange bars indicate the crystal formation energy of pristine, Zn-doped, and Ni-doped $CsPbBr_3$ respectively. (b) Schematic illustration of Ni ion migration from the crystal interior to the surface.

To understand the origin of the improved photostability in individual NPQDs based on this hypothesis, we performed density functional theory (DFT) calculations for orthorhombic crystal structures of pristine, Zn-doped and Ni-doped $CsPbBr_3$. First, to identify the more thermodynamically stable crystal structure, we calculated crystal formation energies for bulk pristine (blue bar in Figure S8a), Zn-doped (green bar in Figure S8a) and Ni-doped $CsPbBr_3$ (orange bar in Figure S8a). The results show that Zn-doped $CsPbBr_3$ exhibits a crystal formation energy comparable to that of pristine $CsPbBr_3$, whereas Ni-doped $CsPbBr_3$ shows a significantly higher formation energy, though all three values remain negative. Thus, all pristine, Zn-doped and

Ni-doped structures are thermodynamically stable; however, we speculate that Ni ions tend to occupy surface positions, as substitution of Ni at internal Pb lattice sites is energetically unfavorable in the bulk crystals as schematically shown in Figure S8b, while Zn ions can more easily incorporate into the lattice. Consequently, we hypothesize that in a nanocrystal, Ni ions at the surface could localize the exciton within the crystal interior, thus reducing interactions between excitons and the surface.

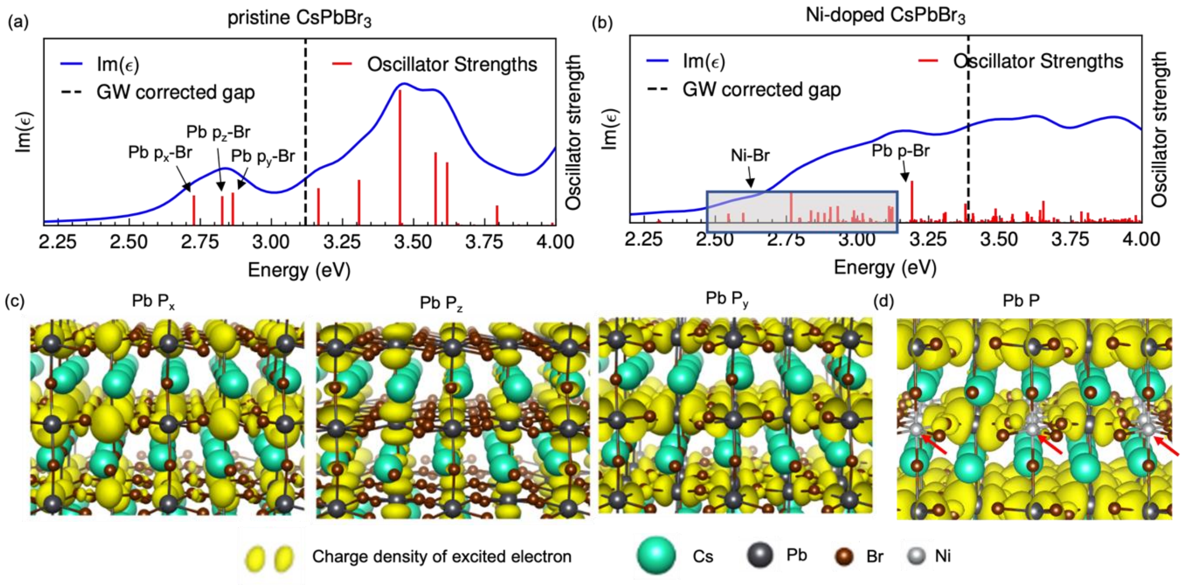

Figure S9. Absorption spectra and charge distributions of excitons. (a, b) Oscillator strength (red vertical bars) and Im(ε) as a function of energy (blue curves). The vertical dashed bars indicate the GW-corrected energy gap. (c, d) Isocharge surfaces of the excited electrons in pristine (c) and Ni-doped (d) $CsPbBr_3$

To further support our hypotheses, it is helpful to investigate the spatial localizations of the excitons for each optical transition. To that end we first need to identify optical transitions originating from the band-edge exciton, which consists of an electron occupying the conduction band minimum bound to a hole occupying the valence band maximum. The emission process in

perovskite quantum dots arises from the radiative relaxation of this band-edge exciton. Technically, we calculate the band structure of electrons in the ground state and account for the corrections of the self-energy of electrons in the crystal within the "G_0W_0" approximation to properly find the electronic band gap [15]. Then we solve the Bethe–Salpeter equation for the interacting electron and hole wave function. This approach allows us to find oscillator strengths (red vertical bars in Figure S9a, b) –probability of photon absorption by an exciton– and imaginary part of the dielectric permittivity of pristine and Ni-doped $CsPbBr_3$ (qualitatively representing the absorption spectra) by adjusting the broadening of each transition line (blue curves in Figure S9a, b). Typically, the transitions from band-edge exciton (either its radiative relaxation or excitation) appear at energies below the bandgap (shown by the black dashed line in Figure S9a, b) so that the three exciton transitions in the pristine $CsPbBr_3$ (Pb $P_{x,y,z}$-Br in Figure S9a) can be interpreted as band-edge exciton transitions. Particularly, in the emission process for pristine $CsPbBr_3$ nanocrystal, these three band-edge exciton transitions are well known to originate from bright triplet states, corresponding to contributions from the Pb conduction-band minimum composed of p_x, p_y, and p_z electronic orbitals transitioning to the uppermost Br valence-band (black arrow in Figure S9a)[16]. In contrast, for Ni-doped $CsPbBr_3$, the band-edge exciton transitions split into multiple exciton transitions. These involve the transition of an electron from the Br valence band maximum to the Pb conduction band minimum (as indicated by "Pb p-Br" in Figure S9b and Figure S10b), as well as additional lower-energy transitions of electron from Br valence band to Ni conduction band (grey partially transparent rectangle indicated by "Ni-Br" in Figure S9b and grey arrow in Figure S10b). Notice that despite the emergence of "Ni-Br" transitions in the calculations, we anticipate that such transitions are not captured by our experiments. We assume that it is primarily because Ni atoms lack a well-defined periodic arrangement in the crystal lattice due to (i) their tendency to

migrate toward the surface, as previously discussed in Figure S8b and (ii) a relatively low Ni/Pb ratio in the synthesized sample. Consistently with this reasoning, we do not detect noticeable lower energy peaks associated with "Ni-Br" transitions in either the absorbance or photoluminescence spectra of the Ni-doped $CsPbBr_3$ nanocrystal solution. Therefore, we attribute the experimentally observed emission to the "Pb p-Br" transition, where the "Ni-Br" transitions appear to be negligible. Consequently, we identify that the dominant band-edge exciton transition in both pristine and Ni-doped $CsPbBr_3$ remains the "Pb p-Br" transition and we hypothesize that Ni-doping influences the spatial localization of the band-edge exciton associated with the "Pb p-Br" transition.

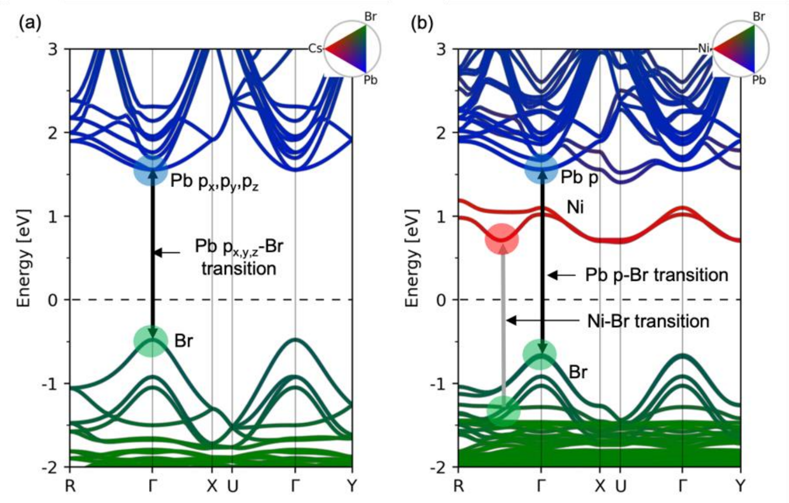

Figure S10. Band structures of electrons in the ground state in pristine and Ni-doped $CsPbBr_3$. (a) The band structure of pristine $CsPbBr_3$. The black arrow indicates the transition from Pb p_x, p_y, p_z orbitals energy level (highlighted by the blue partially transparent circle) to Br orbitals energy level (highlighted by the green partially transparent circle). (b) The band structure of Ni-doped $CsPbBr_3$. The black arrow indicates the transition from the Pb P orbitals energy level to the Br orbitals energy

level. The grey solid arrow indicates the transition from the Br orbitals' energy levels to the Ni orbital energy levels.

To visualize the spatial localization of the excitonic states, we plotted the charge density distributions of excited electrons as a function of coordinates (represented as yellow isocharge surfaces in Figure S9c, d). In these charge density plots, holes' positions are fixed around Br atoms for each band-edge exciton transition. For pristine $CsPbBr_3$, the excited electron charge densities corresponding to all three Pb $P_{x,y,z}$-Br band-edge transitions appear highly delocalized. We guess that such delocalization could expose excitons to the nanocrystal surface. In contrast, for Ni-doped $CsPbBr_3$, the charge density of the excited electron in the conduction band originating from Pb p orbital states, remains delocalized throughout the crystal but notably excludes regions near Ni atoms (grey sphere in Figure S9d). Combining our previous arguments —that Ni ions prefer surface sites and excitons remain isolated from Ni— we infer that the exciton arising from the Pb p orbital would become spatially localized within the nanocrystal, thereby reducing exciton-surface interactions and enhancing stability.

Summarizing our theoretical analysis, we see that although the origin of the observed photostability enhancement can still be under debate, our DFT calculations provide a hint that spatial localization of the exciton in the presence of Ni atoms can be a path to improve photostability by reducing interaction with the environment.

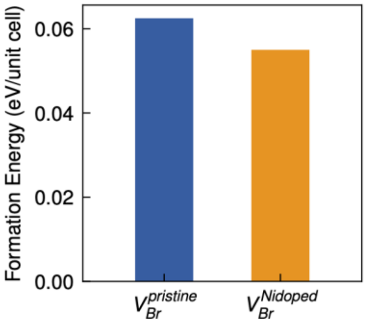

Figure S11. Defect formation energies of bromide vacancy in pristine $CsPbBr_3$ (left) and Ni doped $CsPbBr_3$ (right)

Additionally, we calculated the defect formation energies for both pristine and Ni-doped $CsPbBr_3$ to evaluate whether Ni incorporation reduces defect formation (Fig. S11). The DFT results show that the formation energy of the bromide vacancies is comparable, or even slightly lower, in Ni-doped $CsPbBr_3$ compared to pristine $CsPbBr_3$. This indicates that Ni doping does not lead to

reduced defect formation. Therefore, we conclude that the enhanced photostability observed in Ni-doped $CsPbBr_3$ is not related to the defect passivation.

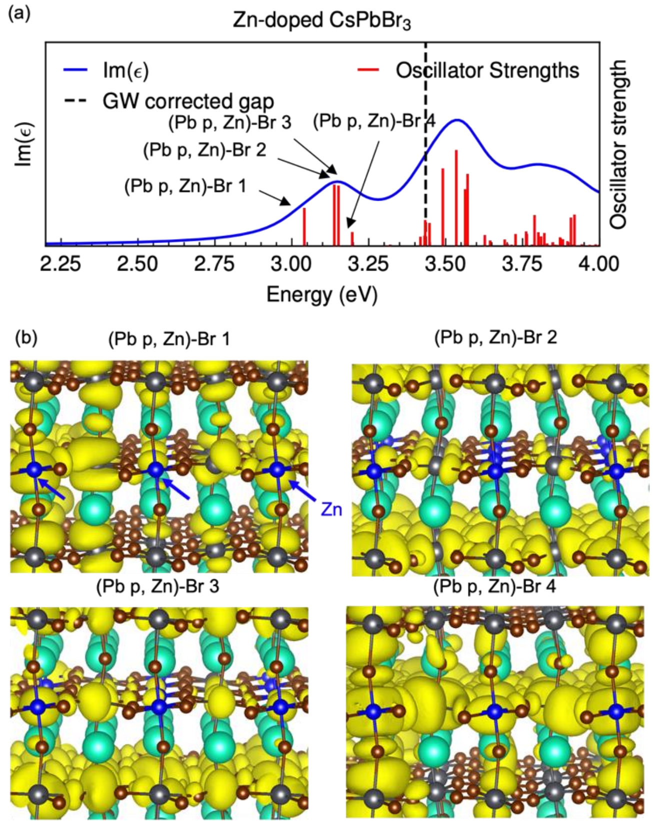

Figure S12. Absorption spectra and charge distributions of excitons in Zn-doped $CsPbBr_3$. (a) Oscillator strength (red vertical bars) and Im(ϵ) as a function of energy (blue curves). The vertical dashed bars indicate the GW-corrected energy gap. (b) Isocharge surfaces of the excited electrons in Zn-doped $CsPbBr_3$

To clarify the mechanistic origin of the superior stability of Ni-doped PQDs compared to Zn-doped PQDs, we calculate the absorption spectra (Fig. S12a) and spatial distribution of electrons (Fig. S12b) for Zn-doped $CsPbBr_3$, in direct comparison with Ni-doped $CsPbBr_3$ (Fig. S9b, d). Zn-doped $CsPbBr_3$ has four band-edge exciton transitions, involving the transition of an electron from the Br valence band maximum to the Pb or Zn conduction band minimum (as indicated by "(Pb p,

Zn)-Br" in Figure S9c). The spatial distribution of electrons of Ni-doped (Fig. S9d) and Zn-doped $CsPbBr_3$ (Fig. S12b) reveals that electrons are spatially separated from the Ni ions, leading to their localization within the nanocrystal (Fig. S9d). In contrast, Zn doping allows the spatial distribution of electrons near Zn ions (Fig. S12b). Therefore, the enhanced photostability in Ni-doped $CsPbBr_3$ arises from this spatial localization of the carrier, which does not apply to Zn-doped $CsPbBr_3$, highlighting a fundamentally different stabilization behavior.

References

1. Dudarev, S. L., Botton, G. A., Savrasov, S. Y., Humphreys, C. J. & Sutton, A. P. Electron-energy-loss spectra and the structural stability of nickel oxide: An LSDA+U study. *Phys. Rev. B* **57**, 1505–1509 (1998).
2. Sander, T., Maggio, E. & Kresse, G. Beyond the Tamm-Dancoff approximation for extended systems using exact diagonalization. *Phys. Rev. B* **92**, 045209 (2015).
3. Dancoff, S. M. Non-Adiabatic Meson Theory of Nuclear Forces. *Phys. Rev.* **78**, 382–385 (1950).
4. Krieg, F. *et al.* Monodisperse Long-Chain Sulfobetaine-Capped CsPbBr3 Nanocrystals and Their Superfluorescent Assemblies. *ACS Cent. Sci.* **7**, 135–144 (2021).
5. Zhu, C. *et al.* Room-Temperature, Highly Pure Single-Photon Sources from All-Inorganic Lead Halide Perovskite Quantum Dots. *Nano Lett.* **22**, 3751–3760 (2022).
6. Igarashi, H., Yamauchi, M. & Masuo, S. Correlation between Single-Photon Emission and Size of Cesium Lead Bromide Perovskite Nanocrystals. *J. Phys. Chem. Lett.* **14**, 2441–2447 (2023).
7. Pierini, S. *et al.* Highly Photostable Perovskite Nanocubes: Toward Integrated Single Photon Sources Based on Tapered Nanofibers. *ACS Photonics* **7**, 2265–2272 (2020).

8. D'Amato, M., Tan, Q. Y., Glorieux, Q., Bramati, A. & Soci, C. Color-Tunable Mixed-Cation Perovskite Single Photon Emitters. *ACS Photonics* **10**, 197–205 (2023).

9. Morad, V. *et al.* Designer phospholipid capping ligands for soft metal halide nanocrystals. *Nature* **626**, 542–548 (2024).

10. D'Amato, M. *et al.* Highly Photostable Zn-Treated Halide Perovskite Nanocrystals for Efficient Single Photon Generation. *Nano Lett.* **23**, 10228–10235 (2023).

11. Rainò, G. *et al.* Underestimated Effect of a Polymer Matrix on the Light Emission of Single CsPbBr3 Nanocrystals. *Nano Lett.* **19**, 3648–3653 (2019).

12. Moon, H., Lee, C., Lee, W., Kim, J. & Chae, H. Stability of Quantum Dots, Quantum Dot Films, and Quantum Dot Light-Emitting Diodes for Display Applications. *Advanced Materials* **31**, 1804294 (2019).

13. Aristidou, N. *et al.* The Role of Oxygen in the Degradation of Methylammonium Lead Trihalide Perovskite Photoactive Layers. *Angewandte Chemie International Edition* **54**, 8208–8212 (2015).

14. Yuan, G. *et al.* The Degradation and Blinking of Single CsPbI3 Perovskite Quantum Dots. *J. Phys. Chem. C* **122**, 13407–13415 (2018).

15. Onida, G., Reining, L. & Rubio, A. Electronic excitations: density-functional versus many-body Green's-function approaches. *Rev. Mod. Phys.* **74**, 601–659 (2002).

16. Becker, M. A. *et al.* Bright triplet excitons in caesium lead halide perovskites. *Nature* **553**, 189–193 (2018).
